# Targetable IDH1 mutation identified in a rare case of pancreatic serous cystadenocarcinoma but not a series of serous cystadenomas

**DOI:** 10.1093/jscr/rjac096

**Published:** 2022-03-22

**Authors:** Yuxi Zhang, Autumn Hammonds, Karen Tran-Harding, Kurt B Schaberg, Rashmi T Nair, Chi Wang, Yuanyuan Wu, Prakash K Pandalai, Jill Kolesar, Joseph Kim, Michael J Cavnar

**Affiliations:** Department of Surgery, University of Kentucky, Lexington, KY, USA; Department of Pathology, University of Kentucky, Lexington, KY, USA; Department of Radiology, University of Kentucky, Lexington, KY, USA; Department of Pathology, University of Kentucky, Lexington, KY, USA; Department of Radiology, University of Kentucky, Lexington, KY, USA; Markey Cancer Center, University of Kentucky, Lexington, KY, USA; Markey Cancer Center, University of Kentucky, Lexington, KY, USA; Department of Surgery, University of Kentucky, Lexington, KY, USA; College of Pharmacy, University of Kentucky, Lexington, KY, USA; Department of Surgery, University of Kentucky, Lexington, KY, USA; Department of Surgery, University of Kentucky, Lexington, KY, USA

## Abstract

Serous cystadenocarcinoma (SCAC) of the pancreas is rare, with only 35 cases reported in the literature. We present a case of SCAC, comparing the clinical presentation, management and molecular features of this case to a series of serous cystadenoma (SCA), which may be a precursor. Compared with SCAs (*n* = 5), SCAC was larger (11.5 vs median 3.6 cm). The case of SCAC invaded the spleen and exhibited distant metastasis, a requirement for diagnosis since pathologic features are otherwise indistinguishable from SCA. *VHL* mutations have been reported in about half of SCA in the literature. Accordingly, we identified either somatic or germline *VHL* mutations in 3 of 5 SCAs (60%), yet no pathogenic mutation was identified in the SCAC. A somatic mutation in *IDH1* was found in SCAC only. This has been associated with multiple malignancies, is targetable with the drug ivosidenib and should be studied as a progression factor in SCAC.

## INTRODUCTION

Pancreatic cystic neoplasms are divided into four categories: serous cystic neoplasms (SCN), mucinous cystic neoplasms (MCN), intraductal papillary mucinous neoplasms (IPMN) and solid pseudopapillary neoplasms (SPN) [1]. SCN of the pancreas is a well-recognized, largely benign class of pancreatic cystic neoplasms [1]. Nevertheless, serous cystadenoma (SCA) may have the potential to progress to serous cystadenocarcinoma (SCAC), a rare diagnosis first described in 1989 [2]. To date, 35 malignant pancreatic SCN cases (15 with distant metastases) have been reported worldwide [3]. A recent systematic review showed overall favorable outcome for SCAC with 77% (17/22) alive at a median follow-up of 2 years [4]. Definitive diagnosis of SCAC compared with SCA is challenging given similar clinical, radiographic and even pathologic findings with SCA, with a key distinguishing feature being distant metastasis [4–6].

In addition to the analysis of cyst fluid CEA and amylase, molecular markers have been applied to categorize pancreatic cystic neoplasms. Alterations in *VHL* and *GNAS* appear unique to SCA and IPMN, respectively, while mutations in *KRAS* and *RNF43* are associated with both IPMNs and MCN [1], and alterations in *CTNNB1* may be seen in SPN and IPMN [1]. Current guidelines do not support mutational analysis for SCN diagnosis but recommend such testing if the diagnosis of IPMN or MCN is unclear [7]. To date, genomic analysis of SCAC has not been reported. It is unknown whether SCA is a precursor lesion to SCAC, and factors associated with progression from SCA to SCAC are unknown. One potential molecular marker for SCAC is the *VHL* gene mutation, which has been reported in somatic SCA samples [1, 8]. *VHL* is an autosomal dominant tumor suppressor gene, and mutations are associated with von Hippel–Lindau (VHL) syndrome, a complex syndrome that includes cysts in multiple organs, such as the pancreas and the kidney, and other neoplasms [9]. Here, we present a case of pancreatic SCAC, with retrospective comparison of clinical and molecular data to five SCA using an institutional review board-approved protocol.

## CASES REPORT/SERIES

**Figure 1:**
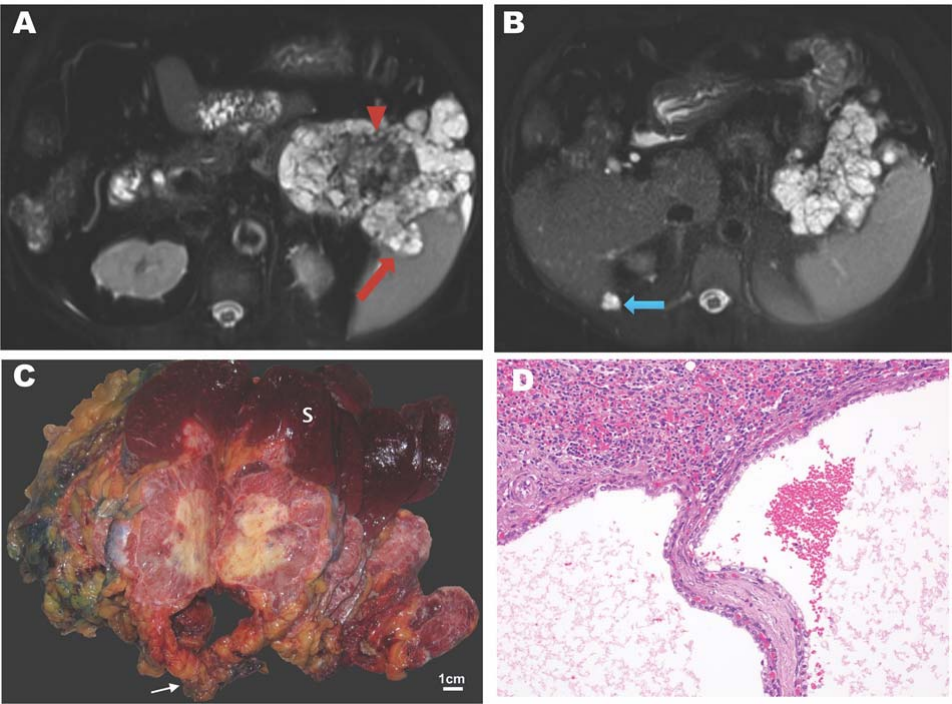
Imaging and pathology of serous cystadenocarcinoma of the pancreas. (**A**) *T*_2_-weighted MR image showing microcysic lesion with invasion into spleen (red arrow) and central dark scar (arrowhead) with (**B**) peritoneal lesion at inferior tip of the liver (blue arrow). (**C**) Gross specimen photograph demonstrating the large pancreatic mass (white arrow) with invasion into the spleen (S) and central necrosis (scale bar, 1 cm). (**D**) Photomicrograph demonstrating invasion of the splenic parenchyma (superior, H&E, 20×).

A 74-year-old woman with a history of hypertension, hypercholesterolemia and gastroesophageal reflux presented with dyspnea and uncontrolled hypertension. Her past surgical history included wrist surgery and a cholecystectomy. Family history was notable for leukemia, lung, extrahepatic bile duct and colon cancer. Other symptoms included early satiety and increased gastroesophageal reflux. Pulmonary embolism was initially suspected, but computed tomography instead showed a 10 cm, multi-cystic, partially calcified mass in the tail of pancreas. Percutaneous biopsy was performed revealing an SCN**.** Magnetic resonance (MR) imaging revealed an 11.5-cm lesion in the pancreatic body and tail. On *T*_2_-weighted images, a microcystic mass with a bosselated surface and a central dark scar (Fig. 1A) was seen. Malignancy was suspected on the basis of invasion into the splenic parenchyma and a cystic lesion adjacent to the liver with similar features (Fig. 1B).

The patient underwent a distal subtotal pancreatectomy/splenectomy and resection of the cystic lesion adjacent to the liver in 2018. Surgical pathology revealed an 11.5 × 10.0 × 9.0 cm mass that largely replaced the pancreatic body and tail. This mass had a pinkish, glistening, solid and cystic cut surface with central chalky white necrosis (Fig. 1C). This lesion was grossly adherent to the splenic hilum. The surgical margins were widely uninvolved by the tumor. Microscopically, the tumor had the classic morphology of a serous cystic neoplasm of the pancreas with cysts lined by cuboidal to flattened cells with bland, central, round nuclei and abundant clear cytoplasm. PAS stains demonstrated abundant glycogen. There were large areas of coagulative necrosis, and the tumor focally invaded the splenic parenchyma grossly (Fig. 1C) and microscopically (Fig. 1D). Mitotic figures were not identified. The separately submitted perihepatic nodule showed identical morphology, consistent with a distant metastasis. Altogether, these findings were diagnostic of a serous cystadenocarcinoma. An outside consultation was obtained from the Johns Hopkins Department of Pathology which confirmed the diagnosis.

Post-operatively, the patient had a prolonged hospital course and multiple readmissions related to a post-operative pancreatic fistula. She was found to have pancreas divisum, and her management required an interventional radiology drain, an endoscopic cyst-gastrostomy and an endoscopic retrograde cholangio-pancreatography with placement of a stent in her dorsal and ventral pancreatic ducts; 32 months post-operatively, she was clinically well and without evidence of disease.

The SCAC case was compared with five archival cases of SCA resected from 2016 to 2019, identified from an institutional pathology database. Among these six total patients with SCN of the pancreas, the majority were female (Table 1). None carried a clinical diagnosis or family a history of VHL syndrome. Compared with the 5 SCA patients, the SCAC patient was slightly older (74 vs median 70 years old) at surgery. Compared with the resected SCAs, the SCAC was larger (11.5 vs median 3.8 cm). The SCAC and 3 SCA were resected for symptoms, while the other 2 SCA were resected for indeterminate imaging findings. The SCAC spanned the body and tail of pancreas and the locations of the 5 SCAs were as follows: 2 tail, 1 neck, 1 body and 1 head. Distant metastasis was only observed in the SCAC as a small peritoneal nodule adjacent to the liver. Benign splenic simple cysts and multiple benign multiple peritoneal mesothelial inclusion cysts were noted in two SCAs, respectively. The majority of patients underwent distal pancreatectomy/splenectomy (SCAC and 4 out of 5 SCAs). All patients were alive and without recurrence at last follow-up with a median follow-up period of 12 months after surgery.

**Table 1:** Characteristics of patients with SCN

**Lesion**	**Age**	**Sex**	**Tumor** **size (cm)**	**Symptoms**	**Location**	**Invasion and/or Distant metastasis**	**Procedure** ^**A**^	**Follow-up (months)**	**Pathogenic somatic mutations**			**Pathogenic germline mutations**
									*CTNNB1, GNAS, KRAS, RNF43*	*VHL*	*IDH1*	*VHL*
SCAC	74	F	11.5	Early satiety, reflux, dyspnea	Body and tail	Spleen, peritoneum	DP/S	32	−	−	+	−
SCA^C^	58	F	3.4	Abdominal pain, nausea	Tail	None	DP/S	8	−	+	−	−
SCA	72	F	2.6	Abdominal pain	Tail	None	DP/S	16	−	−	−	−
SCA^D^	75	M	3.6	None^B^	neck	None	DP/S	35	−	−	−	+
SCA	68	F	3.9	None^B^	body	None (one splenic benign simple cyst)	DP/S	4	−	+	−	−
SCA	59	F	9.5	jaundice, pruritis, loose stools	Head	None (multiple benign mesothelial inclusion cyst)	PD	7	−	−	−	−

Symbols used in the table: −, no pathogenic mutations detected; +, pathogenic mutations detected.

^A^DP/S, distal pancreatectomy/splenectomy; PD, pancreaticoduodenectomy.

^B^Patients with definitive radiographic findings of SCA typically do not undergo resection, rather patients with an indeterminate or concerning lesion (such as concern for intraductal papillary mucinous neoplasm with high-risk features) may undergo resection with final pathology showing SCA.

^C^Patient did not show clinical signs of VHL syndrome.

^D^This patient had a history of a simple renal cyst and bilateral peripelvic cysts but did not have clinical VHL syndrome.

Somatic Whole Exome Sequencing (WES) was performed on the resected specimens. No mutations were seen in *KRAS*, *GNAS*, *RNF43* or *CTNNB1* as previously described in non-serous cystic pancreatic lesions [1, 8]. *VHL* mutations (N78S, S68S?, L63 and S80R) were found in 4 SCAs and another (PYPTL97) in the SCAC (Table 1). One of these mutations in an SCA was labeled clinically pathogenic by ClinVar [10], a database annotating observed clinical phenotype with genomic variants, while another was designated as pathogenic using Functional Analysis through Hidden Markov Models (FATHMM), an algorithm for predicting pathogenicity of a mutation [11]. The remaining *VHL* mutations were designated as non-pathogenic variants including that in SCAC. Loss of heterozygosity (LOH) of chromosome 3p (which contains the *VHL* gene) was previously reported in SCA [1, 8]; however, none of our SCA or SCAC samples showed LOH in chromosome 3. A Clinvar pathogenic mutation in *IDH1* (R132H) was seen in SCAC only, while no other pathogenic *IDH1* mutations were found by Clinvar or FATHMM in the other specimens. *IDH1* is an oncogene encoding isocitrate dehydrogenase 1, a component of NADPH metabolism, with mutations reported in glioma, cholangiocarcinoma and acute myeloid leukemia [12]. Germline WES was also performed, focusing on *VHL* and *IDH1.* One SCA patient had a Clinvar pathogenic germline *VHL* mutation that has been observed in VHL syndrome [13], although this patient did not exhibit clinical features of VHL syndrome. There were no germline pathogenic *IDH1* mutations. In summary, only 3 of 5 (60%) of SCA exhibited either somatic or germline pathogenic *VHL* mutations, while SCAC had none, and a pathogenic somatic *IDH1* mutation was exclusive to SCAC.

## DISCUSSION

Definitive diagnosis of SCAC is difficult, as it was in this case. Diagnosis of SCAC was confirmed only after pathology confirmed similar histologic features from the primary tumor and the distant peritoneal metastasis. This diagnostic dilemma is seen across the literature. Imaging findings (CT or MR) or pathological results are unlikely to distinguish primary malignant SCN from benign SCN given their similar pathological and radiological presentations unless clear invasion or metastasis is seen [4–6]. About 25% of reported SCACs in the literature were initially diagnosed as benign SCN by surgical pathology and were subsequently designated as cystadenocarcinoma only when metachronous metastases were identified, suggesting the possible need for post-resection surveillance of some SCN [6]. A systematic review noted that the median time interval from diagnosis of SCN to metachronous metastases was 3 years (range 1–10) with a median primary tumor size of 10.5 cm (range 5–14) for those who developed metastases [4]. Thus, it is reasonable to offer post-resection surveillance for large SCN. Since the minimum size of SCAC reported in literature was 4 cm [4], for patients not being offered surgery for presumed benign SCA, ongoing surveillance could be considered for SCN with a size of at least 4 cm based on limited retrospective evidence. For patients undergoing non-operative surveillance, surgery is recommended if symptoms appear or if there is concerns for malignant conversion [7].

Surgical resection for patients with malignant SCN should be considered in patients with good functional status and operable disease status. For patients with inoperable disease, evidence for chemotherapy or radiation is limited. Of the 35 cases reported in literature, one patient with inoperable, stage IV serous cystadenocarcinoma was reported to receive a 4-month course of neoadjuvant chemotherapy with no response, although the patient had minimal progression during this time demonstrating the relatively indolent biology [3, 5]. Chemotherapy in combination with radiation therapy reduced tumor size and allowed for surgery in two cases [14]. Likewise, it is also unknown whether adjuvant chemotherapy is beneficial after resection of SCAC. In our case, we recommended close surveillance, and the patient remains disease-free after 32 months.

Consistent with the current literature, our 6 SCNs did not demonstrate mutations in *KRAS*, *GNAS*, *RNF43* or *CTNNB1* as described in non-serous cystic pancreatic lesions [1, 8]. Pathogenic somatic or germline *VHL* mutations were found in 3 of 5 SCAs (60%) but not in SCAC. Presence of *VHL* mutations may be helpful but not required for diagnosis of SCA, since VHL mutations are seen in only approximately half of the confirmed SCAs [1, 8]. None of our SCAC or SCA samples showed evidence of LOH in chromosome 3, as previously reported in some but not all SCA [1, 8]. Thus, neither *VHL* mutation nor LOH of chromosome 3 (which would alter *VHL* gene function) was necessary for SCAC development in our patient. The missense mutation in *IDH1* (R132H) is noted in OncoKB for its oncogene status [12] and may serve as a genomic marker for SCAC. The *IDH1*-inhibitor ivosidenib has been FDA-approved for treatment of acute myelogenous leukemia and advanced cholangiocarcinoma [15]. This drug might have the potential to treat *IDH1*-mutant inoperable or recurrent SCAC, and thus, further study of this gene as a progression factor in SCAC is warranted.

In conclusion, we report a rare case of serous cystadenocarcinoma. Clinically, radiologically and pathologically distinguishing SCAC from SCA is difficult, with definitive diagnosis requiring synchronous or metachronous metastasis or organ invasion. Pathogenic *VHL* mutations were seen in some of SCA, but not SCAC. Further study is needed to determine whether this gene (or alterations thereof via LOH) plays a role in pathogenesis of SCN. A targetable pathogenic somatic mutation in the oncogene *IDH1* was unique to SCAC and warrants further study as a progression factor.
